# Evaluation of Mis-Selection of End Vertebrae and Its Effect on Measuring Cobb Angle and Curve Length in Adolescent Idiopathic Scoliosis

**DOI:** 10.3390/jcm13154562

**Published:** 2024-08-05

**Authors:** José Hurtado-Avilés, Vicente J. León-Muñoz, Fernando Santonja-Medina, Paolo Raimondi, Francisco Martínez-Martínez

**Affiliations:** 1 Sports & Musculoskeletal System Research Group (RAQUIS), University of Murcia, Avda. Buenavista 32, El Palmar, 30120 Murcia, Spain; joseaviles@um.es (J.H.-A.); fernando@santonjatrauma.es (F.S.-M.); 2 Department of Orthopaedic Surgery and Traumatology, Hospital General Universitario Reina Sofía, Avda. Intendente Jorge Palacios, 1, 30003 Murcia, Spain; 3 Instituto de Cirugía Avanzada de la Rodilla (ICAR), C/Barítono Marcos Redondo 1, 30005 Murcia, Spain; 4 Department of Surgery, Paediatrics and Obstetrics & Gynaecology, Faculty of Medicine, University of Murcia, Avda. Buenavista 32, El Palmar, 30120 Murcia, Spain; fmtnez@gmail.com; 5 Department of Orthopaedic Surgery and Traumatology, Hospital Clínico Universitario Virgen de la Arrixaca, Ctra. Madrid-Cartagena, s/n, El Palmar, 30120 Murcia, Spain; 6 Già Prof. Associated at the Department of Applied Clinical and Biotechnological Sciences, University of L’Aquila, Palazzo Camponeschi, Piazza Santa Margherita 2, 67100 L’Aquila, Italy; paoloraimondi1942@gmail.com

**Keywords:** adolescent idiopathic scoliosis, Cobb angle, measurement errors, radiographic assessment, spine curvature

## Abstract

**Background:** The Cobb angle is critical in assessing adolescent idiopathic scoliosis (AIS) patients. This study aimed to evaluate the error in selecting the upper- and lower-end vertebrae on AIS digital X-rays by experienced and novice observers and its correlation with the error in measuring the Cobb angle and determining the length of the scoliotic curves. **Methods:** Using the TraumaMeter v.873 software, eight raters independently evaluated 68 scoliotic curves. **Results:** The error percentage in the upper-end vertebra selection was higher than for the lower-end vertebra (44.7%, CI95% 41.05–48.3 compared to 35%, CI95% 29.7–40.4). The mean bias error (MBE) was 0.45 (CI95% 0.38–0.52) for the upper-end vertebra and 0.35 (CI% 0.69–0.91) for the lower-end vertebra. The percentage of errors in the choice of the end vertebrae was lower for the experienced than for the novices. There was a positive correlation (r = 0.673, *p* = 0.000) between the error in selecting the end vertebrae and determining the length of the scoliotic curves. **Conclusions:** We can conclude that errors in selecting end vertebrae are common among experienced and novice observers, with a greater error frequency for the upper-end vertebrae. Contrary to the consensus, the accuracy of determining the length of the scoliotic curve is limited by the Cobb method’s reliance on the correct selection of the end vertebrae.

## 1. Introduction

Adolescent idiopathic scoliosis (AIS) is a complex three-dimensional misalignment of the spine consisting of a coronal curve of more than 10°, vertebral rotation [1] and altered sagittal curvature [2,3,4], with no congenital or neuromuscular abnormal findings [5]. AIS can progress over the years if untreated, especially during growth, and can cause significant musculoskeletal problems, pain in adulthood, pulmonary impairment and psychological problems [6,7,8,9]. Measuring the Cobb angle on a standing posteroanterior full-length spine X-ray is the gold standard for diagnosing AIS, assessing its severity, monitoring its changes and making decisions about its treatment [10,11,12,13,14]. The selection of the end vertebrae plays a pivotal role in determining the Cobb angle and accurately describing the curve’s length, which is crucial for defining appropriate treatment strategies. For instance, if a scoliotic curve exceeds 50°, posterior spinal fusion can be the most appropriate treatment in some cases, which irreversibly alters the biomechanical function of the spine [15,16,17]. In this surgical procedure, selecting the appropriate end vertebrae determines the curve length to be fused, affecting the biomechanical stability achieved and reducing the incidence of post-surgical complications [18,19]. Conversely, there is a consensus in the literature that selecting the end vertebrae in the Cobb angle measurement method in AIS X-ray is a significant source of intrinsic error [20,21,22,23] or the primary source of intrinsic error [24,25,26,27]. The literature on the accuracy and precision of the Cobb method in AIS X-ray indicates that potential sources of intrinsic error in traditional manual measurement include the erroneous choice of vertebral endplates, inaccurate drawing of lines along the vertebral endplates, imprecise drawing of perpendicular lines and inaccurate angle measurement itself [22,25,26,27,28,29]. Within the random error of the measurement method, we can differentiate the extrinsic and the intrinsic error. The intrinsic error is the part of the random, aleatory or unpredictable error attributable to the measuring instruments, the equipment or the procedure itself.

This study aims to evaluate the error in selecting the upper- and lower-end vertebrae on AIS X-ray images by experienced and novice observers. Additionally, it aims to explore the correlation between the error in selecting the end vertebrae and the error in measuring the Cobb angle and the correlation between the error in selecting the end vertebrae and the error in quantifying the length of scoliotic curves.

## 2. Materials and Methods

### 2.1. Measurement Tool

**Figure 1:**
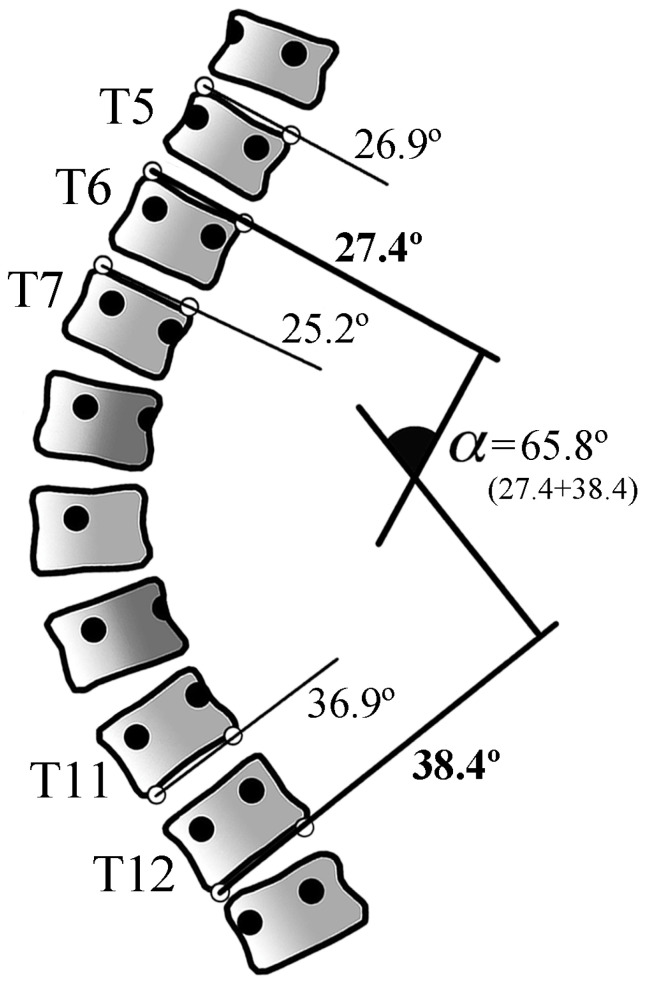
Schematic description of the end vertebrae selection with the software Traumameter v.873. Lines along the several vertebral endplates can be drawn when there is doubt about which ones are more tilted. The software will automatically choose the vertebrae that are most inclined to the horizontal (in this example, T6 (27.4°) and T12 (38.4°)). α: Cobb angle.

The software Traumameter v.873 [Hurtado-Avilés and Santonja-Medina, registration number 08/2021/374, Murcia, Spain] was employed to identify the end vertebrae, measure the Cobb angle and quantify the length of the scoliotic curves. This software replicates the manual Cobb angle measurement method observed in AIS X-rays, enabling measurements with high intra- and inter-observer accuracy and precision (MBE = 1.8°, SD = 0.65°, CI95% 1.58°–2.02° and MBE = 1.82°, SD = 0.59°, CI95% 1.62°–2.02°, respectively) [30]. The software eliminates the intrinsic error due to inaccurate drawing of perpendicular lines and inaccurate angle measurement. On the other hand, it reduces the error due to the wrong choice of the end vertebrae and inaccurate line drawing along the vertebral endplates through tools such as the ability to zoom in on the regions of interest and to vary the contrast (fractional difference in the optical density of brightness between two regions of an image) of the digital X-ray image. The software allows the observer to draw lines along the vertebral endplates of various upper- and lower-end vertebrae and selects the steepest ones, returning the Cobb angle result in degrees (Figure 1). Our group has published research on the software’s accuracy in previous studies [30].

### 2.2. Study Design and Measurement Protocol

A prospective and observational study was conducted on 68 scoliotic curves in 42 standing frontal full-length spine X-rays of patients with AIS. The X-ray images were selected retrospectively from an image repository during routine medical care of patients with AIS. Our study adhered to the World Medical Association Declaration of Helsinki’s ethical standards, as revised in 2013. This study was exempted from the requirement for ethical approval since the complete and irreversible anonymisation of the images did not involve data processing. The X-ray sample was homogeneous, had equivalent image quality and was without defects. The X-ray images were obtained natively in digital format (in DICOM, with a resolution of 283.46 pixels/mm).

According to the angular classification proposed by the International Society on Scoliosis Orthopaedic and Rehabilitation Treatment [31], the selected X-rays showed asymmetry in 2 cases (curves between 0° and 10°), low scoliosis in 17 cases (curves between 11° and 20°), moderate scoliosis in 25 cases (curves between 21° and 35°), moderate-to-severe scoliosis in 9 cases (curves between 36° and 40°), severe scoliosis in 11 cases (curves between 41° and 50°), severe-to-very-severe scoliosis in 1 cases (curves between 51° and 55°) and very severe scoliosis in 3 cases (curves with 56° or more). Of the upper-end vertebrae, 66 (97.1%) were thoracic and 2 (2.9%) were lumbar. Of the lower-end vertebrae, 35 (51.5%) were thoracic and 33 (48.5%) were lumbar.

Hopkins absolute reliability criteria [32,33] were employed to assess validity and reliability. These criteria stipulate that a minimum of 30 cases must be included, at least six blinded observers must act as assessors and at least three tests must be conducted per observer, with a minimum of two-week intervals between tests. A specialist in orthopaedics and traumatology and a specialist in physical medicine and rehabilitation, with over 35 years of professional experience each in the field of the spine, were engaged in a joint and simultaneous measurement of all scoliotic curves on the same computer to establish a gold standard for the Cobb angles, the end vertebrae and the length of each curve.

Both experienced observers measured each radiograph three times on the same computer using the TraumaMeter v.873 software. These two specialists also have extensive experience with the software (one of them has participated in its development as a consultant). The accuracy and precision of their measurements were examined with the software and found to be less than 1° for the Cobb angle measurements. Using the method explained above, it is possible to control the internal consistency and temporal stability of the measurements obtained, which determine the random error in establishing the final vertebrae. This resolution is not affected by significant variations in reliability or accuracy. On the other hand, the systematic error or bias in the estimation will also be reduced when the measurements are made jointly by the two experienced observers (i.e., the results have a consensus that avoids tendencies to overestimate or underestimate the measurements).

The research was conducted with eight independent evaluators with varying experience levels in measuring Cobb angles. Four observers, designated as “Experienced”, were one orthopaedic specialist and three physical therapy and rehabilitation specialists with more than 20 years of experience in the routine measurement of radiographs of scoliotic patients in their daily practice, but who had not determined their intra-observer error. Despite their theoretical knowledge of X-ray measurement techniques for the spine, the four “Novice” observers from various health sciences disciplines (without being orthopaedic surgeons) had never applied Cobb’s method in practice. Prior to the commencement of the measurements, a five-hour briefing was held, during which comprehensive information was provided on the study and training in the use of the Traumameter v.873 software. In each X-ray, each observer identified the curves, measured Cobb’s angle on them with the software and recorded the resulting measurement and the uppermost and lowest vertebrae of each scoliotic curve in an Excel table. The observers conducted the measurements on three occasions, with a one-month interval between each measurement. A total of 1632 curves were evaluated for this study (204 curves by each observer). The study coordinator randomly assigned the sequence in which the radiographic images were presented in each test to avoid bias, keeping the randomisation key confidential.

### 2.3. Statistics

Statistical analysis was performed using the Statistical Package for the Social Sciences (SPSS), version 25 for Windows (SPSS, Inc., Chicago, IL, USA). The values of the variable “Cobb angle error” (eCobb) were obtained in degrees with one decimal place due to the scale of the software measuring tool. The values of the variables “error in the choice of the upper or cranial end vertebra” (eCr), “error in the choice of the lower or caudal end vertebra” (eCa) and “error in the quantification of the length of the scoliotic curve” (eLC), were obtained in the unit “number of vertebrae per curve” with one decimal place. The eCobb, eCr, eCa and eLC values for each scoliotic curve were obtained from the distribution of the 24 scoliotic curve measurements (8 observers in the three tests). In the measurement distributions of each curve, values lower than Q1 − (1.5xIQR) and higher than Q3 + (1.5xIQR) (where IQR is the interquartile range) were identified. These values were considered outliers and eliminated from each distribution (1.96% of eCobb; 0.12% of eCr; 0.06% of eCa; 0% of eLC). The percentage error of each observer in the choice of the upper- and lower-end vertebrae was used to obtain four distributions, namely “percentage of upper and lower end vertebrae wrongly chosen by experienced and novices, respectively”. These distributions did not contain any outliers. The Shapiro–Wilk test was employed to ascertain that the *p*-values of the data from these distributions were above the significance level of 0.05, with the null hypothesis that the data fit a normal distribution being accepted. In all cases, *p* ≥ 0.34. All variables were reported as Mean Bias Error (MBE), Standard Deviation (SD), Standard Error of the Mean (SEM) and Confidence Interval of 95%. A Student’s *t*-test was employed to ascertain whether the differences in MBE values between each pair of measurements were statistically significant. Two-sided *p* values and the CI95% were reported, and significance was accepted at *p* < 0.05. Pearson correlation was obtained between the distributions eCr + Ca (sum of the distributions eCr and eCa) and eCobb and between the distributions eCr + Ca and eLC.

## 3. Results

Table S1a,b (Supplementary Material) show the gold standard and observer-recorded data for the Cobb angle and end vertebrae, respectively. Table S2 (Supplementary Material) shows the outliers removed. Table 1 shows the data for the error selection of the end vertebrae, the measurement of the Cobb angle and the quantification of the length of the scoliotic curves for the total set of measurements.

The values of eCobb (Cobb angle error) are given in degrees, and those of eCr (error in the choice of the upper or cranial end vertebra), eCa (error in the choice of the lower or caudal end vertebra), eCr + eCa and eLC (error in the quantification of the length of the scoliotic curve) are given in the number of vertebrae per scoliotic curve. MBE is Mean Bias Error. SD is Standard Deviation. SEM is Standard Error of the Mean. IC95% is Confidence Interval of 95%.

**Figure 2:**
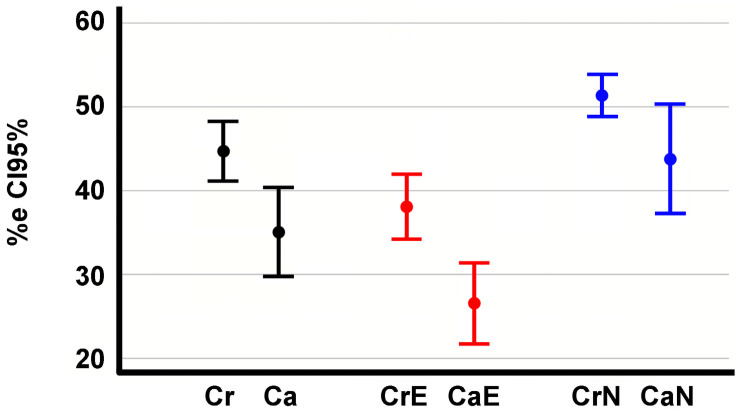
95% confidence intervals of the percentage error in the choice of upper or cranial and lower or caudal end vertebrae by Experienced observers (CrE, CaE, respectively) and upper- or cranial- and lower- or caudal-end vertebrae by Novice observers (CrN, CaN, respectively).

The error rate in the choice of the upper-end vertebrae is higher than for lower-end vertebrae (44.7%, CI95% 41.05–48.3 compared to 35%, CI95% 29.7–40.4). This difference is statistically significant (*p* = 0.008) (Figure 2). The error percentage in the choice of the end vertebrae is lower in the experienced than in the novices, with a statistically significant difference (*p* = 0.009 between upper-end vertebrae, *p* = 0.000 between lower-end vertebrae) (Figure 2). The percentage of erroneous choices made by the experienced evaluators was 37.99% (CI95% 33.96–42), while that of novices was 51.35% (CI95% 48.77–53.97). Experienced evaluators incorrectly identified the lower-end vertebra in 26.35% (CI95% 21.45–31.25) of the measurements, while novices incorrectly identified it in 43.75% (CI95% 37.06–50.44).

Experienced evaluators incorrectly identified one end vertebra in 36.6% (274) of the scoliotic curves, while in 15.4% (126) of the cases, they incorrectly identified two end vertebrae. By contrast, novices incorrectly identified one end vertebra in 34.7% (278) of the scoliotic curves, while in 30.39% (248) of the cases, they incorrectly identified two end vertebrae.

Table S3 (Supplementary Material) presents the number of incorrectly selected end vertebrae and the error in the Cobb angle for each measurement. It demonstrates that the error in the Cobb angle measurements for each scoliotic curve is randomly distributed and unrelated to the number of incorrectly selected end vertebrae.

Further analysis was performed on the distribution of Cobb angle measurements for each scoliotic curve to identify the 50% of measurements closest to the gold standard value. This analysis showed that 44.6% (365) of these measurements were carried out with the two end vertebrae correctly identified. By contrast, 33.9% (278) were obtained with one of the end vertebrae incorrectly identified, and 21.5% (176) were obtained with both end vertebrae incorrectly identified.

**Figure 3:**
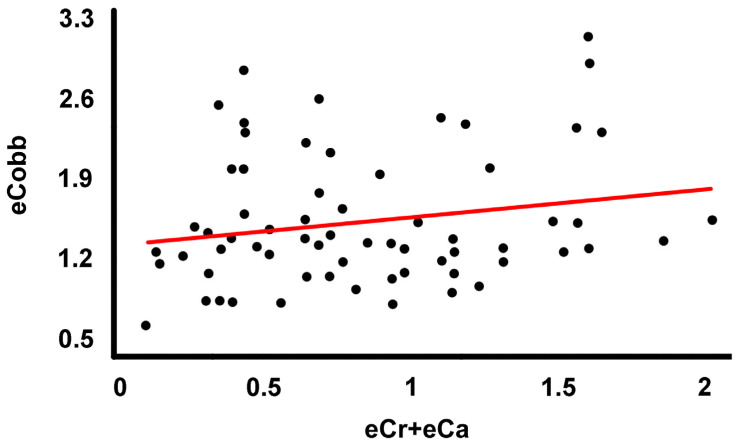
Regression line fitted to the point cloud corresponding to the variables “number of end vertebrae incorrectly selected in a scoliotic curve” (eCr + eCa) and “error in measurement of the Cobb angle” (eCobb) in each curve.

**Figure 4:**
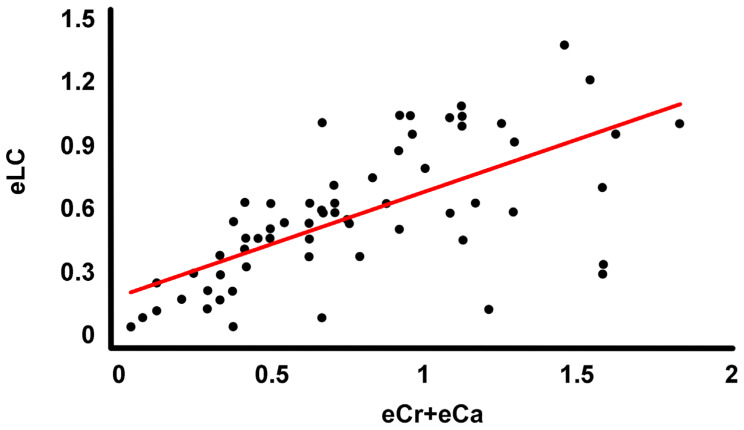
Regression line fitted to the point cloud corresponding to the variables “number of incorrectly selected end vertebrae in the curve” (eCr + eCa) and “error in determining the length of the scoliotic curve” (eLC).

The correlation between the error in the choice of the end vertebrae and the error in the Cobb angle measurements is not statistically significant (r = 0.198, r^2^ = 0.039, *p* = 0.111) (Figure 3). The correlation between the error in the choice of the end vertebrae and the error in the determination of the length of the scoliotic curves is statistically significant (r = 0.673, r^2^ = 0.453, *p* = 0.000) and positive (Figure 4). The linear equation forced to the origin of coordinates describing the correlation is eCr + eCa = 1.243·eLC (r = 0.610).

## 4. Discussion

The most important finding of this study was that errors in selecting end vertebrae are common among both experienced and novice observers, with a greater frequency of error in the upper-end vertebrae. Likewise, the lack of dependence between the end vertebrae error and the Cobb angle error is also relevant.

In AIS, the error in selecting the uppermost and the lowest vertebrae when measuring the Cobb angle can be relevant to the correct decision on treatment (observation, physiotherapy, orthosis, surgery) and its characteristics [10,11,13,34]. For example, if the applicable treatment for a scoliotic curve is instrumented arthrodesis, a longer-than-necessary fusion means sacrificing too many segments of motion and, therefore, function [34,35,36,37], increased risk of neurological complications and infection and increased cost. There is consensus that the incorrect selection of the end vertebrae is a significant source of intrinsic error in measuring the Cobb angle on AIS X-rays [22,23,27]. However, in studies of Cobb angle inaccuracy where the error is measured using preselected end vertebrae, the erratum is not significantly improved, with intra-observer MBE in such studies ranging from 3° to 9.04° [25,26,28,29,37].

Artificial intelligence will likely play an essential role in Cobb angle measurement in the coming years. There is a growing number of publications on deep learning methods for Cobb angle measurement [38]. Not all of the proposed systems are more accurate than human operators [39], but there is a trend towards a progressive increase in the accuracy of such systems [40,41].

Our study’s absolute error in the Cobb angle mean was MBE = 1.53°, CI95% 1.42°–1.63°. With this accuracy and precision, we found that the error in the Cobb angle measurements was not related to the correct choice of the end vertebrae (*p* = 0.111). Of the 50% of Cobb angle measurements closest to the actual value, 55.4% (454) were obtained by incorrectly selecting at least one end vertebrae. In summary, the erroneous choice of the end vertebrae is not a source of intrinsic error in the Cobb method but a consequence of the inaccurate drawing of the lines along the vertebral endplates.

The error in selecting the end vertebrae and the error in measuring the Cobb angle have the exact cause when using the Traumameter v.873 software. When using the manual Cobb method, the sources of error are many, but with our software, they are reduced to one; the inaccuracy in drawing the straight line parallel to the upper and lower surface of the end vertebrae is greater than the difference in inclination of the vertebrae adjacent to the end vertebrae and the end vertebrae. Due to the high accuracy and precision of the TraumaMeter v.873 software, the error in the choice of the end vertebrae results in a minimal error in determining the Cobb angle, as the difference in inclination between the correct and incorrectly chosen end vertebrae is tiniest. Our study found that the error rate in choosing the end vertebrae is higher in upper-end vertebrae (44.7%) than in lower-end vertebrae (35%), which is statistically significant. The fact that upper-end vertebrae tend to appear in the thoracic region, with the overlapping of different organs and structures on X-ray, or better visibility of the lower region of the endplate architecture [42], could justify the more significant error in their choice.

The error rate in choosing end vertebrae was statistically significantly higher in novice observers than in experienced observers. Experienced observers wrongly chose at least one of the end vertebrae in 52% of the measurements (400) and novices in 65.1% (526). This aspect can be explained by the lower Cobb angle measurement error shown by experienced measurers [30] when measuring with the TraumaMeter v.873 software and manually traditionally. Our results show a significant positive correlation between the error in choosing end vertebrae and the error in determining the length of the scoliotic curves. From the regression line obtained from the correlation, we can predict an increase in the error in scoliotic curve length determination of 0.805 vertebrae for each incorrectly chosen end vertebra. The error in determining the end vertebrae is due to a limitation of the Cobb method itself. Its systematic error is, in many cases, greater than that required to select the end vertebrae correctly.

This study is not without its limitations. Firstly, we did not consider the computer equipment of each observer (e.g., visible image size, display resolution, luminance, contrast ratio or the characteristics of the mouse or touchpad), which may have influenced the accuracy of the measurements. Secondly, the outliers removed from the distribution used in the study could be due not only to imperfect measurement but also to errors in recording the value of the measurements in the database provided by each observer. Thirdly, we lacked the necessary radiographic projections to present the curve patterns according to, for example, Lenke’s classification [43], which may have helped explain the significant error in selecting the upper-end vertebra. Despite these limitations, the authors believe that the study results are valuable. One of the strengths of our study is that its design meets the Hopkins criteria (minimum of 30 cases, at least six blinded observers and at least three tests per observer, separated by at least two weeks) [32,33]. Also, we established training sessions for the observers to avoid measurement bias.

The clinical importance cannot be overstated, as the correct determination of the end vertebrae can significantly influence therapeutic decisions, such as the length of the arthrodesis in the surgical treatment of AIS. It is crucial to note that the error usually made by observers in misidentifying the cranial and caudal end vertebrae on AIS X-ray does not change the value of the Cobb angle measurements in a statistically significant way. However, such an error leads to the misjudgement of the length of the scoliotic curves, which has clinical implications.

## 5. Conclusions

Errors in selecting end vertebrae are common among experienced and novice observers, with a greater frequency of error in the upper-end vertebrae. There is no correlation between the error in selecting the end vertebrae and the Cobb angle error. Inappropriate choice of end vertebrae leads to an estimated error in determining the length of the scoliotic curves of 0.805 vertebrae for each end vertebra chosen incorrectly.

## Figures and Tables

**Table 1:** Statistical values of each error distribution for the total set of measurements.

	eCr	eCa	eCr + eCa	eCobb	eLC
MBE	0.45	0.35	0.80	1.53	0.43
SD	0.28	0.27	0.47	0.44	0.34
*n*	68	68	68	65	66
SEM	0.03	0.03	0.06	0.05	0.04
IC95%	0.38–0.52	0.29–0.42	0.69–0.91	1.42–1.63	0.35–0.51

## Data Availability

The data supporting the conclusions of this article will be made available by the authors on request.

## Supplementary Materials

The following supporting information can be downloaded at https://www.mdpi.com/article/10.3390/jcm13154562/s1, Table S1a: gold standard and observer-recorded data for the Cobb angle. Table S1b: gold standard and observer-recorded data for end vertebrae. Table S2: outliers removed. Table S3: number of incorrectly selected end vertebrae and the error in the Cobb angle for each measurement.
